# Laryngeal Anatomy and Morphometry: Foundations for Interdisciplinary Collaboration and Personalized Management of Laryngeal Pathology

**DOI:** 10.3390/diagnostics16172739

**Published:** 2026-08-26

**Authors:** Anca Simioniuc-Petrescu, Mihai Dumitru, Adrian Costache, Daniela Vrinceanu, Andreea Marinescu, Nicoleta Sanda, Alina Lavinia Antoaneta Oancea, Adina Zamfir Chiru Anton, Romica Cergan

**Affiliations:** 1 ENT Department, Carol Davila University of Medicine and Pharmacy, 020021 Bucharest, Romania; anca.simioniuc@yahoo.com (A.S.-P.); orldumitrumihai@yahoo.com (M.D.); vrinceanudana@yahoo.com (D.V.); 2 Pathology Department, Carol Davila University of Medicine and Pharmacy, 020021 Bucharest, Romania; 3 Imaging Department, Carol Davila University of Medicine and Pharmacy, 020021 Bucharest, Romania; andreea_marinescu2003@yahoo.com; 4 General Surgery Department, Carol Davila University of Medicine and Pharmacy, 020021 Bucharest, Romania; 5 ENT Department, Elias Clinical Hospital, Carol Davila University of Medicine and Pharmacy, 011461 Bucharest, Romania; dr.alina.oancea@gmail.com; 6 ENT Department, Grigore Alexandrescu Clinical Hospital, Carol Davila University of Medicine and Pharmacy, 011743 Bucharest, Romania; zamfiradina@yahoo.com; 7 Anatomy Department, Carol Davila University of Medicine and Pharmacy, 020021 Bucharest, Romania; r.cergan@gmail.com

**Keywords:** laryngeal morphometry, precision laryngology, laryngeal cancer staging, artificial intelligence segmentation, imaging

## Abstract

Laryngeal pathology requires individualized management because the larynx integrates airway protection, phonation, swallowing, and respiratory function within a compact and highly variable anatomical framework. This narrative review examines how laryngeal anatomy and morphometry support interdisciplinary collaboration and personalized care in oncologic, stenotic, functional, and reconstructive laryngeal disease. Evidence from morphometric studies, CT, MRI, endoscopy, ultrasonography, three-dimensional reconstruction, artificial intelligence, and multidisciplinary clinical workflows was synthesized qualitatively. Key parameters—including vocal fold length, glottic width, thyroid cartilage angle, cricoid diameter, subglottic diameter, anterior commissure thickness, and the status of paraglottic and pre-epiglottic spaces—provide actionable information for diagnosis, T-staging, airway assessment, surgical planning, reconstruction, and functional rehabilitation. Morphometry concretely informs clinical decisions: thyroid cartilage angle guides thyroplasty and phonosurgical planning; subglottic diameter supports stenosis surgery and airway instrumentation; and anterior commissure, conus elasticus, cartilage, and deep-space measurements refine oncologic staging and margin strategy. Technological accelerators, including AI segmentation, radiomics, 3D printing, photogrammetry, and ultrasonography, extend morphometry from static measurement toward predictive modeling and patient-specific simulation. However, implementation remains limited by heterogeneous CT protocols, inconsistent measurement planes, uneven access to advanced technologies, lack of global normative databases, and unresolved ethical issues surrounding AI validation and data governance. This review supports standardizing CT morphometry using parallel vocal fold planes, routinely measuring anterior commissure thickness in T1 glottic cancer, incorporating ultrasonography as a first-line morphometric tool in voice clinics, and validating AI segmentation against population-specific morphometric norms. Laryngeal morphometry should therefore become a routine decision-making framework for precision laryngology.

## 1. Introduction

The human larynx is a complex organ situated at the intersection of the respiratory and digestive tracts, serving the vital functions of airway protection [1,2]. In recent decades, the management of laryngeal pathologies—ranging from malignant neoplasms to benign stenoses and neurological disorders—has shifted from standardized protocols toward a paradigm of personalized medicine [1]. This transition is driven by the recognition that laryngeal anatomy is highly variable, influenced by factors such as sexual dimorphism, age-related remodeling, and pathological changes [3,4].

Traditional surgical approaches often relied on generalized anatomical models, which could lead to suboptimal functional outcomes, particularly regarding voice quality and airway patency [5]. However, the emergence of precision tools such as 3D reconstruction, biomedical engineering, and artificial intelligence has allowed for a more granular understanding of individual laryngeal geometry [6,7]. In this review, interdisciplinary collaboration is justified only insofar as it is grounded in shared morphometric information: measurable laryngeal parameters provide the common reference language through which different specialists interpret tumor extension, airway size, cartilage involvement, reconstructive requirements, and expected functional consequences [8,9].

Laryngeal anatomy has evolved from a purely descriptive foundation for diagnosis into a functional, imaging-based, and predictive tool. This perspective is essential for effective staging, treatment selection, surgical planning, the preservation of vocal function, and post-therapeutic monitoring. The conceptual framework of this review reflects the transition from descriptive anatomy to functional, predictive morphometry. In contemporary laryngology, anatomical knowledge is not limited to structural identification; it is increasingly essential for guiding imaging interpretation, tumor staging, airway management, surgical planning, reconstructive strategies, and functional rehabilitation. By adopting a global assessment approach, clinicians can better address the interplay between respiratory, phonatory, and deglutition-related morbidities [10,11].

This review aims to synthesize the role of laryngeal anatomy and morphometry as foundations for interdisciplinary collaboration by identifying the specific measurements that connect radiologic interpretation, surgical planning, anesthetic airway management, oncologic staging, pathological margin assessment, rehabilitation, and emerging technological workflows.

## 2. Methodology

This narrative review synthesizes current data on the role of laryngeal anatomy and morphometry in the personalized management of laryngeal pathology. We examine literature encompassing anatomical structures, morphometric assessment via CT, MRI, and endoscopy, and clinical applications in oncology, phonosurgery, and laryngotracheal stenosis, as well as emerging insights from artificial intelligence and three-dimensional reconstruction. Furthermore, we evaluate how the precise integration of high-definition imaging and functional physiological assessments facilitates a shift toward organ-preserving strategies in oncological care [12,13,14]. By synthesizing multidisciplinary data from oncologists, surgeons, and speech-language pathologists, this review highlights how anatomical precision directly influences treatment selection and the mitigation of functional morbidity [1,15].

This narrative review was conducted through a comprehensive and non-systematic search of peer-reviewed literature to synthesize current evidence on the interdisciplinary management of laryngeal diseases. The methodology focuses on the integration of anatomical morphometry, advanced imaging, and emerging technologies like artificial intelligence in clinical practice.

To improve reproducibility, the final search was completed on 1st June 2026. Database-specific search algorithms were applied as follows: PubMed/MEDLINE: ((“larynx”[MeSH Terms] OR laryngeal OR glottic OR subglottic OR supraglottic) AND (morphometry OR morphometric OR anatomy OR “vocal fold length” OR “thyroid cartilage angle” OR “cricoid diameter” OR “subglottic diameter”) AND (“computed tomography” OR CT OR “magnetic resonance imaging” OR MRI OR endoscopy OR ultrasonography OR “3D reconstruction” OR “3D printing” OR “artificial intelligence” OR “deep learning”)); Scopus: TITLE-ABS-KEY((laryngeal OR larynx OR glottic OR subglottic OR supraglottic) AND (morphometry OR anatomy OR “vocal fold length” OR “thyroid cartilage angle” OR “subglottic diameter”) AND (CT OR “computed tomography” OR MRI OR endoscopy OR ultrasonography OR “three-dimensional reconstruction” OR “artificial intelligence”)); Web of Science: TS=((laryngeal OR larynx OR glottic OR subglottic OR supraglottic) AND (morphometry OR anatomy OR “vocal fold length” OR “thyroid cartilage angle” OR “cricoid diameter” OR “subglottic diameter”) AND (CT OR MRI OR endoscopy OR ultrasonography OR “3D reconstruction” OR “3D printing” OR “artificial intelligence” OR radiomics)); and Cochrane Library: (laryngeal OR larynx OR glottic OR subglottic OR supraglottic) AND (morphometry OR anatomy OR imaging OR stenosis OR cancer) AND (CT OR MRI OR endoscopy OR ultrasonography OR reconstruction). Boolean combinations used AND to link the main concepts of laryngeal anatomy/morphometry, imaging or technology, and clinical application, while OR was used within each concept group to capture synonyms and related terms. Filters were limited to human studies, English-language publications or articles with reliable English translation, peer-reviewed articles, reviews, clinical guidelines, and publications from January 2000 to December 2025; no restrictions were applied regarding study design during initial screening, although editorials, conference abstracts without full text, and poorly documented case reports were excluded during eligibility assessment.

The 2000–2025 restriction was selected to prioritize literature generated during the period in which cross-sectional imaging, digital morphometry, three-dimensional reconstruction, endoscopic visualization, artificial intelligence, and patient-specific surgical planning became clinically relevant and methodologically comparable. This cutoff is therefore most directly justified for technology-dependent evidence, where earlier studies often used acquisition protocols, reconstruction methods, or reporting standards that are not readily comparable with contemporary CT, MRI, ultrasound, photogrammetry, radiomics, and AI-based workflows. However, the authors acknowledge that anatomical and cadaveric morphometric evidence did not begin in 2000 and that foundational pre-2000 studies may remain relevant for basic laryngeal anatomy. For this reason, the date restriction was not applied as an absolute exclusion criterion for historically important anatomical concepts; older foundational sources were considered when they provided indispensable anatomical definitions, established terminology, or morphometric principles not superseded by later evidence. The main synthesis nevertheless emphasizes post-2000 studies because the objective of this review is to connect laryngeal anatomy with contemporary precision imaging, interdisciplinary decision-making, and emerging personalized technologies rather than to provide a complete historical account of descriptive laryngeal anatomy.

Reporting of this narrative review was guided by the Scale for the Assessment of Narrative Review Articles (SANRA), with attention to the justification of the review’s importance, clear statement of aims, description of the literature search and selection strategy, appropriate referencing, balanced synthesis of evidence, and transparent discussion of limitations. Because the manuscript is a narrative rather than a systematic review, SANRA was used as reporting guidance rather than as a formal protocol for evidence grading or meta-analytic assessment.

Study Selection and Inclusion Criteria

Study selection was performed in two sequential stages. First, titles and abstracts retrieved from each database were screened independently by two reviewers to identify publications potentially relevant to laryngeal anatomy, morphometry, imaging, technological applications, and interdisciplinary management. Second, the full texts of potentially eligible articles were assessed separately against the predefined inclusion and exclusion criteria. Disagreements at either stage were resolved through discussion between the reviewers; when consensus could not be reached, a third author reviewed the article and made the final inclusion decision. Duplicate records were removed before screening, and uncertainties regarding eligibility were resolved conservatively by advancing the article to full-text assessment rather than excluding it at the title/abstract stage.

Studies were eligible for inclusion if they provided quantitative morphometric data, discussed interdisciplinary workflows (e.g., MDT approaches), or evaluated the diagnostic accuracy of technological interventions. Inclusion criteria favored original research, including cadaveric studies and clinical trials investigating laryngeal dimensions and surgical outcomes; technological evaluations, including reports on the feasibility and accuracy of AI-assisted endoscopy and 3D-printed laryngeal prostheses; clinical guidelines, including national and international protocols for laryngeal cancer and stenosis management; and articles published in English or those with high-quality English translations [16,17,18,19].

Case reports and series were included only when they described novel surgical techniques or unique interdisciplinary challenges, such as complex airway management during total laryngectomy [20,21].

Data Extraction and Synthesis

Data were extracted from the selected literature with a focus on the synergy between diagnostic parameters and therapeutic execution. For morphometric studies, key values such as the anteroposterior diameter and glottic width were compared across different demographic groups to highlight sexual dimorphism [3,4]. For AI applications, performance metrics including sensitivity (71–98%) and specificity (86–98%) were synthesized to assess the readiness of these tools for clinical adoption [6,22]. The findings were qualitatively integrated into thematic sections—Anatomy, Imaging, Surgical Collaboration, and Future Perspectives—to provide a holistic view of personalized laryngeal care [1,23].

Specifically, the implementation of advanced diagnostic modalities, such as high-resolution head and neck magnetic resonance imaging, offers superior soft-tissue contrast and precision in delineating submucosal tumor spread and subsite-specific invasion, thereby critically optimizing preoperative surgical planning for conservative approaches [14,24,25]. However, the reliance on subjective parameters like vocal fold motility continues to introduce diagnostic variability, highlighting the urgent need for standardized imaging protocols and reproducible morphometric metrics [26,27]. A comprehensive understanding of laryngeal anatomy is essential for the accurate interpretation of clinical symptoms and diagnostic assessments. While the glottis and true vocal folds are central to phonation, the supraglottic and subglottic regions are of primary clinical significance in the management of tumoral, stenotic, and respiratory pathologies. Furthermore, the thyroid, cricoid, and arytenoid cartilages form the essential supporting architecture of the laryngeal complex; consequently, any structural alterations in these cartilages can profoundly impact both airway patency and vocal quality. Beyond these macro-structural considerations, recent research underscores significant sexual dimorphism and age-related remodeling within these cartilaginous frameworks, which are critical for establishing reliable normative reference values in clinical morphometry [3,28]. Furthermore, the clinical application of multiparametric MRI—utilizing sequences like T2-weighted imaging, diffusion-weighted imaging, and contrast-enhanced T1—enables an objective differentiation between neoplastic infiltration, peritumoral inflammation, and mass effects, particularly within the posterior paralaryngeal space [29].

Laryngeal morphometry shifts the focus of descriptive anatomy toward a framework of objective measurements, facilitating accurate diagnosis, staging, therapeutic planning, and ongoing patient monitoring. Key parameters—including vocal fold length, glottic width, cricoid diameter, subglottic dimensions, and the thyroid cartilage angle—serve as fundamental variables that shape both clinical presentation and therapeutic decision-making. Furthermore, the utilization of multiplanar reformation in cross-sectional imaging allows for the alignment of scan planes parallel to the vocal folds, a critical adjustment that minimizes the anatomical distortions often caused by the inherent obliquity and dynamic nature of laryngeal structures [30,31]. Advanced imaging techniques, such as dynamic phonation maneuvers during CT or MRI, further refine these measurements by capturing the vocal folds in both abducted and adducted states [32]. These sophisticated imaging strategies address the inherent limitations of lateral radiography, where overlapping soft tissues and obscure landmarks frequently compromise the accuracy of vocal fold length estimation [33]. Furthermore, sex-based structural divergence, particularly regarding thyroid lamina dimensions and cricoid diameters, necessitates the application of population-specific normative databases to avoid misinterpretations in clinical practice [4,34].

Laryngeal morphometry provides the quantitative anatomical framework necessary for personalized diagnosis and the development of patient-specific therapeutic interventions. By standardizing measurements of key laryngeal structures, clinicians can better account for individual variability driven by sexual dimorphism, age, and pathological remodeling [3,4].

## 3. Results

The literature search revealed that laryngeal morphometry and technological integration are the primary drivers of diagnostic and therapeutic precision.

### 3.1. Core Morphometric Parameters

The vocal folds exhibit significant sexual dimorphism, with males generally possessing longer and wider folds than females [4,34]. Research indicates that the dimension of the membranous glottis is a reliable “calibration distance” for assessing the absolute area and length of glottal lesions during endoscopic evaluation [33]. There is also a strong positive correlation between the anteroposterior diameter of the subglottic area and the VFL, suggesting that deeper subglottic structures are predictive of vocal fold dimensions [33]. Recent literature emphasizes that the use of non-parallel CT planes can introduce significant anatomical distortion, undermining the precision required for personalized management [30,31].

The internal angle of the thyroid cartilage is a primary indicator of laryngeal dimorphism, being significantly narrower in males than in females [3,4]. This angle is a critical parameter in thyroplasty and phonosurgery, as it dictates the anterior attachment point of the vocal folds and the overall geometry of the laryngeal skeleton [4,16].

Cricoid and Subglottic Diameter: The cricoid cartilage serves as the only complete ring in the airway, and its sagittal diameter is significantly greater in males [4]. Morphometric data regarding subglottic diameter is essential for managing subglottic stenosis and post-intubation trauma, as it guides the selection of appropriately sized surgical tools and endotracheal tubes [16,33].

### 3.2. Morphometry in Oncologic Staging and Surgical Planning

A personalized surgical plan for laryngeal cancer relies on understanding not just the tumor size, but its relationship to anatomical barriers.

The larynx is divided by fibroelastic membranes, including the conus elasticus and quadrangular membrane, that act as temporary barriers to tumor spread [1]. The pre-epiglottic space and paraglottic space may serve as conduits for occult tumor extension, and understanding the morphometry of these deep spaces via CT or MRI is essential for deciding between partial and total laryngectomy [1,2]. If a tumor crosses the conus elasticus, it frequently invades the cricoid cartilage or subglottis, necessitating a change in the surgical margin approach [35].

Anterior Commissure Thickness: Evaluating the anterior commissure dimension on axial imaging provides a predictive marker for potential tumor infiltration into the thyroid cartilage, as variations in commissural anatomy influence the precision of transoral laser microsurgery [36]. Furthermore, accurate assessment of the subglottic region via high-resolution imaging is vital, as tumor subglottic extension exceeding 15 mm and cricoid cartilage chondrolysis are significant predictors of thyroid gland involvement [37]. Building upon these morphometric insights, clinical integration of head and neck MRI has demonstrated high diagnostic accuracy in mapping tumor invasion across specific laryngeal subsites, significantly improving preoperative staging for total laryngectomy candidates [38]. Moreover, acknowledging substantial shape variation beyond mere size differences allows for more precise patient stratification, as morphotypes can influence the mechanical behavior of the laryngeal skeleton during phonation and airway protection [39].

Furthermore, Broyles’ ligament serves as a recognized weak point in the anterior commissure, where tumor infiltration can compromise the thyroid cartilage, thereby necessitating meticulous preoperative assessment of this anatomical boundary [40].

Morphometric variations are most pronounced when viewing the laryngeal framework across the lifespan, as pediatric anatomy differs significantly from adult standards. In infants, the larynx is positioned more superiorly in the neck—typically at the C3–C4 vertebral level—compared to the more caudal location in adults [19,41]. A critical anatomical distinction involves the airway’s narrowest point; whereas the glottis is the most constricted region in the adult larynx, the subglottis serves as the narrowest point in pediatric patients [19,41]. These developmental differences necessitate specialized morphometric norms for managing pediatric laryngotracheal stenosis, as interventions such as selecting graft sizes for laryngotracheal reconstruction or performing cricotracheal resections rely on these age-specific dimensions [41,42]. Moreover, longitudinal studies utilizing three-dimensional reconstructions reveal that post-pubertal laryngeal shape is influenced by a complex interplay of age, sex, and body mass index, which further modulates the mechanical properties of the vocal apparatus [43]. Additionally, developmental maturation of the hyoid-larynx complex involves progressive ossification of the connections between the central hyoid body and its greater horns, which may manifest as complete bilateral fusion in approximately 25% of adults [44]. Age-related changes, such as ossification of laryngeal cartilages and vocal fold muscle atrophy after the age of 60, further alter the laryngeal framework and must be distinguished from pathological remodeling during imaging interpretation and surgical planning [45,46].

### 3.3. Morphometry Across the Lifespan

The integration of these morphometric values into clinical practice facilitates a shift toward precision laryngology. Quantitative data on thyroid lamina height and width are used to predict vocal fold length, which is fundamental for optimizing outcomes in partial laryngectomies and laryngeal microsurgery [4,16]. Accurate measurements of laryngeal diameters are also required to design virtual and plastic models for preoperative simulation and surgeon training [16].

Morphometric measurements form the basis of biomechanical models that simulate voice production and respiratory airflow [5,34]. In cases requiring reconstruction, such as thyroid cartilage repair, 3D geometrical models derived from CT scans allow for the fabrication of personalized titanium meshes or prostheses tailored to the patient’s unique anatomy [7,45]. While 3D-printed titanium implants represent a significant development, the field is rapidly shifting toward 4D printing. The use of smart polymers, as detailed in the 2023 review ‘Biomaterials-based additive manufacturing for customized bioengineering in management of otolaryngology,’ enables the creation of implants that respond to physiological stimuli—an attribute particularly advantageous given the larynx’s dynamic nature.

Within this lifespan-based framework, understanding age-related changes—such as the atrophy of vocal fold muscle and the calcification of laryngeal cartilages typically seen after age 60—allows clinicians to distinguish between natural structural degeneration and pathological changes [3]. This distinction ensures that treatments for voice disorders are appropriately targeted based on the patient’s biological age and laryngeal morphology [3,16]. Moreover, the prevalence of these calcification patterns, which can reach 54% in adult cohorts, underscores the importance of interpreting imaging studies within the context of normative structural data to avoid diagnostic overestimation [46].

The extent of interindividual variability underscores the necessity for morphometric norms stratified by sex, age, and population-specific characteristics. Consequently, the management of laryngeal pathology cannot be rigidly standardized; instead, it must be tailored to the unique anatomical and clinical profile of each patient. Integrating such data into clinical workflows requires precise protocols, such as venous-phase CT scans to optimize soft-tissue visualization or MRI for assessing laryngeal cartilage involvement in T3 tumors [47]. These personalized models, informed by stereomorphotopometric characteristics, are increasingly critical for developing reconstructive laryngoplasty techniques that account for the unique anatomical constitution of the individual [48]. Moreover, the integration of machine learning algorithms for automated vocal fold segmentation holds the potential to reduce manual diagnostic error, providing clinicians with precise visual metrics that support objective surgical planning and longitudinal monitoring of treatment responses [49]. Emerging research, exemplified by the study ‘A prognostic model integrating radiomics and deep learning based on CT for survival prediction in laryngeal squamous cell carcinoma’, demonstrates that artificial intelligence offers predictive capabilities that extend beyond endoscopic detection, which is pivotal for refining treatment planning. Additionally, the advent of structure-from-motion photogrammetry allows for the high-fidelity reconstruction of laryngeal anatomy directly from endoscopic video, offering a radiation-free alternative for quantitative anatomic assessment in both pediatric and adult populations [20]. Beyond these imaging-based advancements, ultrasonography has emerged as a rapid, non-invasive, and cost-effective modality for defining normative morphological parameters and vocal fold dynamics in clinical settings. This diagnostic utility is further bolstered by recent normative databases that establish essential quantitative references for vocal fold length and dynamics, facilitating a more rigorous framework for the diagnosis of voice disorders and providing a reliable baseline for monitoring pathological progression or the efficacy of interventional therapies [50]. Such standardization is paramount, as it addresses previous clinical limitations and underscores the importance of validating ultrasonography as a robust, objective, and non-invasive modality for patient-centered laryngeal assessment [50].

The multidisciplinary dimension should therefore be understood as measurement-driven rather than role-driven. Laryngeal morphometry supplies the shared anatomical dataset—vocal fold length, glottic width, thyroid cartilage angle, cricoid and subglottic diameters, anterior commissure thickness, cartilage integrity, and deep-space involvement—from which different specialists derive complementary decisions. This framing keeps collaboration connected to the manuscript’s central thread: anatomy and morphometry as clinically actionable foundations.

### 3.4. Technological Extensions of Morphometry

By combining advanced digital modeling from engineering with specialized medical and rehabilitative care, the team ensures that oncologic considerations and patient preferences are balanced to achieve optimal functional reintegration [1,23].

Quantitative studies demonstrate significant sexual dimorphism in selected laryngeal parameters, particularly the internal thyroid cartilage angle and specific laryngeal dimensions, rather than uniformly across all morphometric variables. The internal thyroid cartilage angle is markedly narrower in males compared to females, which contributes to sex-based differences in laryngeal geometry and vocal fold length [3,4]. Computed tomography data shows a strong positive correlation between the anteroposterior diameter of the subglottic area and VFL, indicating that subglottic dimensions can help predict glottic size [33]. Furthermore, age-related changes, such as the ossification of laryngeal cartilages and muscle atrophy, significantly alter the laryngeal framework after the age of 60, impacting both diagnostic interpretation and surgical planning [3].

AI and deep learning models have shown transformative potential in laryngeal endoscopy. Systematic reviews of convolutional neural networks indicate diagnostic sensitivity ranging from 71% to 98% [6,22], diagnostic specificity ranging from 86% to 98% [22], and strong clinical utility in identifying early-stage laryngeal cancer and distinguishing between benign and malignant lesions, thereby serving as an augmentative tool for the ENT surgeon [6,17].

Research into 3D technologies highlights their role in personalized reconstruction. Digital modeling from thin-slice CT scans allows for the creation of patient-specific titanium meshes and prostheses [7]. In cases of thyroid cartilage defect, 3D-printed prostheses manufactured via selective laser melting may improve anatomical fit compared with traditional pre-formed implants [7,45]. However, statements regarding reduced operative time or enhanced laryngeal stability should be interpreted cautiously unless directly demonstrated by comparative clinical evidence in the cited studies; where such evidence is limited, these outcomes should be presented as potential advantages requiring further clinical validation rather than established benefits.

## 4. Discussion

### 4.1. What Morphometry Adds That Descriptive Anatomy Cannot

“Laryngeal anatomy has evolved from a purely descriptive foundation into a functional, imaging-based, predictive tool.” This statement captures the central contribution of morphometry: it transforms anatomical description into measurable, clinically actionable information. Descriptive anatomy identifies structures, whereas morphometry quantifies dimensions, spatial relationships, asymmetry, age-related remodeling, and disease-related distortion. The results of this review underscore that personalized management in laryngology is no longer a theoretical goal but a data-driven reality, because the strong correlation between subglottic and glottic dimensions [33] suggests that preoperative CT morphometry should be a standard component of surgical planning for both laryngeal cancer and stenosis [4,41]. The principal parameters and their direct clinical applications are summarized in Table 1.

The main finding of this review is not merely that multiple morphometric parameters exist, but that their clinical value depends on how consistently they are translated into decisions. Across the included studies, the most actionable measurements were those that connected anatomy to a specific management consequence: subglottic dimensions to airway instrumentation and stenosis reconstruction, anterior commissure morphology to the likelihood of cartilage involvement and margin planning, thyroid cartilage geometry to phonosurgical customization, and deep-space involvement to the feasibility of organ-preserving surgery. This interpretation moves the discussion beyond a repetition of the Results by identifying morphometry as a decision filter: measurements are useful when they reduce uncertainty at a clinically relevant choice point.

### 4.2. How Morphometry Concretely Changes Clinical Decisions

Morphometry changes clinical decisions by linking measurements to specific diagnostic, surgical, anesthetic, oncologic, and rehabilitative choices. The thyroid cartilage angle influences thyroplasty and phonosurgical planning because it affects vocal fold attachment, implant geometry, and the likelihood of postoperative voice optimization [4,16]. Subglottic diameter informs the management of stenosis, the selection of appropriately sized endotracheal tubes, and the design of reconstructive strategies in both pediatric and adult airway surgery [16,33,41]. Accurate measurements of glottic and subglottic dimensions therefore help clinicians anticipate airway resistance, surgical exposure, and the feasibility of organ-preserving interventions [4,33,41]. These measurement-to-decision relationships are organized as proposed surgical decision rules in Table 2.

A second finding is that morphometry does not replace clinical judgment, but changes the level at which judgment is applied. Rather than relying only on qualitative descriptors such as “limited extension,” “possible cartilage involvement,” or “difficult airway,” the reviewed evidence supports a shift toward structured interpretation of measurable thresholds, anatomical planes, and patient-specific geometry. This has practical importance because the same lesion may be staged, exposed, resected, intubated, reconstructed, and rehabilitated differently depending on small variations in glottic width, subglottic diameter, commissural thickness, or cartilage angle. Therefore, the contribution of this review is to underline that precision laryngology begins when morphometric findings are explicitly linked to therapeutic consequences.

Recent evidence on transoral robotic surgery (TORS) should also be considered in this morphometry-driven selection framework for minimally invasive and organ-preserving treatment of supraglottic laryngeal cancer; a 2025 systematic review in Journal of Robotic Surgery reported contemporary operative and functional outcomes, supporting TORS as an additional organ-preserving option when tumor exposure, supraglottic subsite involvement, airway safety, swallowing function, and multidisciplinary expertise are favorable [51].

In oncologic care, morphometry helps determine whether tumor extension respects or crosses key anatomical boundaries, thereby influencing the choice between transoral laser microsurgery, open partial laryngectomy, total laryngectomy, radiotherapy, systemic therapy, or combined treatment [1,2,35]. Precise assessment of the paraglottic and pre-epiglottic spaces, anterior commissure, Broyles’ ligament, conus elasticus, cartilage invasion, and subglottic spread supports individualized staging and margin planning [29,35,36,37,38,40,47]. These key anatomical barriers and their surgical implications are detailed in Table 3. These measurements also explain why collaboration is relevant: radiology, surgery, pathology, anesthesia, oncology, rehabilitation, and engineering do not contribute as independent digressions, but as users of the same morphometric evidence for different clinical decisions.

Patient-reported outcome measures are also essential to clinical decision-making because they link structural preservation to the lived functional consequences of treatment. Tools such as the Voice Handicap Index and Eating Assessment Tool capture the impact of disease and therapy on voice, swallowing, airway protection, and quality of life [9,23]. In this context, multidisciplinary input is relevant when it interprets morphometric and functional data together: for example, persistent glottic insufficiency after partial laryngectomy may be understood through postoperative vocal fold configuration, endoscopic closure pattern, and patient-reported voice handicap, thereby guiding rehabilitation or secondary procedures such as injection laryngoplasty [1,5,9,23].

The authors’ synthesis also suggests that functional outcomes should be interpreted as morphometry-dependent outcomes rather than as separate postoperative endpoints. Voice, swallowing, airway protection, and quality of life are influenced by the extent to which the laryngeal framework, vocal fold geometry, cartilage support, and glottic closure pattern are preserved or reconstructed. This is particularly relevant for organ-preserving approaches, where oncologic adequacy and functional preservation may compete. The discussion therefore emphasizes that morphometric assessment should be paired with patient-reported outcomes and endoscopic functional evaluation, because structural preservation alone does not guarantee meaningful functional recovery.

Table 4 is retained not as a separate inventory of professional roles, but as a synthesis map showing how the same morphometric measurements are translated into specialty-specific decisions. Its purpose is to clarify the foundation of interdisciplinary collaboration by linking each discipline to measurable laryngeal anatomy and to the clinical decisions that depend on those measurements. In this sense, Table 4 explicitly connects the manuscript’s central morphometric evidence to its interdisciplinary framework.

### 4.3. Technological Accelerators of Morphometry

Current development trends indicate a shift from descriptive toward predictive morphometry. Artificial intelligence contributes to this evolution through automatic segmentation of CT and MRI images, recognition of endoscopic lesions, vocal fold segmentation, and correlation of anatomical parameters with functional prognosis [27,49]. Existing reviews and meta-analyses support the potential of AI in the evaluation of laryngeal lesions and early detection, with deep learning models demonstrating sensitivities in identifying laryngeal malignancies reaching up to 98% [6,22,27]. In laryngeal cancer, AI applications based on endoscopic images, videomics, radiomics, and cross-sectional imaging are increasingly positioned as diagnostic support tools rather than replacements for clinical expertise [27,52,62].

Three-dimensional reconstruction, 3D printing, and virtual simulation translate morphometric datasets into practical tools for surgical planning. Digital modeling from thin-slice CT scans enables the creation of patient-specific titanium meshes and customized laryngeal prostheses [5,7,45]. These models address the shared-airway challenge by providing anesthesiologists and surgeons with a precise map of airway geometry, supporting difficult-airway planning and resections that maximize functional preservation [21,55]. This is particularly relevant in phonosurgery and cartilage reconstruction, where millimeter-scale deviations can compromise voice outcomes [5,45].

Ultrasonography and structure-from-motion photogrammetry further expand morphometry beyond conventional CT and MRI. Photogrammetry permits high-fidelity reconstruction of laryngeal anatomy from endoscopic video, offering a radiation-free method for quantitative assessment in pediatric and adult populations [20]. Ultrasonography provides rapid, non-invasive, and cost-effective assessment of vocal fold biomarkers and dynamics, supported by normative data that can help diagnose voice disorders and monitor treatment response [51]. Together, these technologies broaden access to objective laryngeal measurement and longitudinal follow-up [20,51]. The comparative morphometric strengths and clinical utilities of CT, MRI, and ultrasonography are summarized in Table 5.

Another important finding is that the imaging modality should be selected according to the morphometric question being asked. CT appears most useful when bony or cartilaginous geometry, airway caliber, and reproducible linear measurements are required; MRI is most relevant when soft-tissue boundaries, deep-space invasion, and cartilage infiltration are uncertain; and ultrasonography may be most valuable for accessible, repeatable, dynamic assessment of vocal fold parameters. Thus, the discussion does not simply list imaging tools but proposes a task-based imaging hierarchy in which the method is chosen according to the decision that the measurement must support.

### 4.4. Barriers to Implementation

Despite its promise, routine implementation of laryngeal morphometry faces economic, technical, and ethical barriers. The clinical value of 3D-printed titanium meshes and customized laryngeal prostheses is well documented [7,45], yet their integration into routine practice is hindered by the cost of specialized digital modeling, selective laser melting, biocompatible materials, and expert engineering support [7,45]. Technological costs, including 3D printing and AI software, may be particularly restrictive in resource-limited settings. For these interventions to become globally sustainable, manufacturing workflows must be streamlined without compromising the sub-millimeter precision required for laryngeal stability.

Standardization is another major barrier. International standards for laryngeal morphometric measurement are needed to ensure compatibility between institutions, imaging protocols, reporting templates, and research datasets [18]. Without shared landmarks, acquisition parameters, and normative databases, measurements such as glottic width, subglottic diameter, thyroid angle, cartilage invasion, and anterior commissure thickness may vary across centers, reducing reproducibility and limiting clinical adoption [18,30,31,47].

Ethical AI issues also require explicit governance. Deep learning systems can demonstrate strong diagnostic performance [6,22], but the black-box nature of many models complicates clinical accountability when AI outputs conflict with expert judgment [22]. Data privacy, bias in training datasets, ownership of anatomical data, and legal responsibility for AI-assisted decisions must be addressed before these tools can be safely embedded in multidisciplinary care [6,22,61,62]. The transition from experimental research to routine clinical utility therefore requires multicenter validation, transparent reporting, and specialized multimodal models trained on large-scale datasets to ensure reliability and generalizability [53,61].

The review further identifies standardization as the central methodological weakness limiting translation. The findings repeatedly show that morphometry becomes clinically meaningful only when acquisition planes, landmarks, measurement definitions, and reporting templates are comparable across centers. Without this standardization, the same parameter may be measured differently by different teams, weakening its value for staging, operative planning, AI training, and follow-up. The authors therefore interpret protocol harmonization—not only technological innovation—as the most urgent prerequisite for making laryngeal morphometry reproducible and clinically trustworthy.

### 4.5. Future Roadmap

The future roadmap for laryngeal morphometry should prioritize predictive modeling, multimodal AI, and global morphometric databases. Predictive models that combine morphometric measurements with airflow simulation, acoustic analysis, radiomics, deep learning, endoscopic data, histopathology, and patient-reported outcomes could estimate postoperative voice, swallowing, airway, oncologic, and survival outcomes before treatment [5,54,56]. Such tools would improve shared decision-making by helping clinicians compare expected functional and oncologic consequences across surgical and nonsurgical options [1,5,54,56]. A proposed set of AI-driven decision rules is presented in Table 6.

Finally, the authors’ findings indicate that AI should be positioned as an interpretive amplifier rather than as an autonomous decision-maker. AI may improve segmentation, lesion detection, radiomic risk stratification, and workflow integration, but the evidence reviewed does not justify replacing expert multidisciplinary interpretation. The most defensible future use of AI is therefore as a transparent tool that standardizes measurement extraction, flags high-risk anatomical patterns, and supports prospective validation of morphometry-outcome relationships. This framing is important because it prevents overstatement of emerging technologies while still recognizing their potential to transform laryngeal anatomy from a descriptive discipline into a predictive clinical platform.

Multimodal AI should integrate CT, MRI, ultrasonography, endoscopy, photogrammetry, voice analysis, and clinical metadata into transparent decision-support platforms [20,49,51,53,61]. These systems should assist multidisciplinary expertise by offering explainable, auditable recommendations for staging, airway planning, implant design, rehabilitation, and surveillance. In laryngeal trauma and functional impairment, real-time automated analysis of endoscopic and tomographic imagery may also help detect subtle structural lesions, correlate anatomy with function, customize surgical protocols, and guide post-traumatic rehabilitation [57]. The proposed integrated morphometry-with-AI surgical workflow is outlined in Table 7.

Finally, global morphometric databases are needed to establish normative ranges across sex, age, population background, developmental stage, and disease state [3,4,39,41,43,46,51]. Shared international datasets would support standardization, reduce diagnostic variability, improve AI generalizability, and allow laryngeal morphometry to mature from a promising research tool into a reproducible pillar of precision laryngology. Examples of age- and sex-specific morphometric norms relevant to this goal are summarized in Table 8.

## 5. Limitations of the Present Narrative Review

Several limitations should be acknowledged. First, this is a narrative review rather than a systematic review or meta-analysis; therefore, the included literature was synthesized thematically and qualitatively rather than through formal risk-of-bias assessment, pooled effect estimates, or standardized evidence grading. As a result, the conclusions should be interpreted as practice-oriented recommendations derived from converging evidence rather than as definitive guideline-level mandates.

Second, the absence of large, globally representative morphometric databases remains a major limitation for clinical translation. Existing normative data are often restricted to specific populations, age groups, imaging platforms, or cadaveric cohorts, limiting their generalizability across sex, ethnicity, developmental stage, body habitus, and geographic background. This restricts the ability to define universally reliable thresholds for vocal fold length, glottic width, thyroid angle, cricoid diameter, subglottic diameter, anterior commissure thickness, and age-related cartilage remodelling.

Third, heterogeneity in CT acquisition and reconstruction protocols complicates comparison between studies and institutions. Differences in slice thickness, patient positioning, phonatory state, contrast phase, axial plane orientation, and the use of parallel versus non-parallel vocal fold planes can substantially alter measured glottic and subglottic parameters. This variability is particularly relevant when morphometric data are used for T-staging, anterior commissure assessment, subglottic extension measurement, surgical margin planning, or stenosis reconstruction.

Finally, many technological extensions of morphometry, including AI segmentation, radiomics, photogrammetry, ultrasonography, and 3D printing, are still unevenly validated across clinical settings. Their performance may vary according to imaging quality, training datasets, operator expertise, equipment availability, and local workflow integration. Future research should therefore prioritize multicenter validation, standardized acquisition protocols, population-specific reference ranges, transparent AI reporting, and prospective studies linking morphometric measurements to functional, oncologic, and quality-of-life outcomes in the broadest context possible. Future research directions may also be highlighted.

## 6. Final Considerations

As a narrative review, this manuscript should be interpreted as a synthesis of current concepts and converging evidence rather than as a source of definitive guideline-level conclusions. The reviewed literature suggests that laryngeal anatomy is progressively moving beyond descriptive characterization toward clinically useful morphometric interpretation, particularly when anatomical measurements are integrated with imaging, multidisciplinary assessment, and patient-specific treatment planning.

Several practical considerations emerge from this synthesis. CT morphometry may benefit from standardization using parallel vocal fold planes to reduce staging errors caused by oblique acquisition and to improve comparability between institutions. Anterior commissure thickness should be considered during assessment of T1 glottic cancer, because subtle variation in this region may influence the risk of thyroid cartilage involvement, transoral laser microsurgery planning, and the choice between conservative and more extensive treatment. Ultrasonography may also have a growing role as a non-invasive morphometric tool in voice clinics, particularly for assessment of vocal fold length, dynamic biomarkers, and longitudinal monitoring. AI segmentation requires validation against population-specific morphometric norms before automated tools can be reliably used for clinical decision support.

These considerations indicate how morphometry may inform practice without implying that a narrative review alone can establish mandatory clinical rules. Thyroid cartilage angle can support thyroplasty and phonosurgical planning; subglottic diameter can assist stenosis surgery, airway instrumentation, and reconstructive strategy; and measurements of paraglottic, pre-epiglottic, anterior commissure, conus elasticus, and subglottic involvement can contribute to oncologic staging and surgical margin planning. Such applications should be interpreted within multidisciplinary decision-making and adapted to patient-specific anatomy, function, comorbidity, disease extent, and available institutional expertise.

Overall, the future development of precision laryngology appears to depend on standardized imaging protocols, validated normative databases, accessible ultrasonographic workflows, transparent AI systems, and prospective studies linking morphometric measurements to functional and oncologic outcomes. When interpreted by multidisciplinary teams, 3D reconstruction, 3D printing, predictive modelling, and quantitative morphometry may support earlier diagnosis, safer airway management, more accurate staging, individualized reconstruction, and better preservation of voice, swallowing, and quality of life. These final considerations therefore frame morphometry as a promising decision-support framework that requires further validation, rather than as a definitive conclusion drawn from a systematic evidence hierarchy.

## Figures and Tables

**Table 1:** Key Laryngeal Morphometric Parameters and Their Clinical Applications.

Morphometric Parameter	Quantitative/Anatomical Insight	Clinical Application
Subglottic Anteroposterior (AP) Diameter [33]	Strong positive correlation with vocal fold length (VFL)	Predicts glottic size; improves planning for partial laryngectomy and stenosis surgery
Internal Thyroid Cartilage Angle [3,4]	Narrower in males; major determinant of sexual dimorphism	Critical for thyroplasty, phonosurgery, and predicting VFL
Subglottic Extension (>15 mm) [37]	Threshold associated with thyroid gland involvement	Guides decision between partial vs. total laryngectomy; predicts need for thyroidectomy
Anterior Commissure Thickness [36]	Variation influences risk of thyroid cartilage infiltration	Determines suitability and margin planning for transoral laser microsurgery

**Table 2:** Proposal of Surgical Decision Rules Based on Morphometry.

Morphometric Criterion	Threshold/Key Measurement	Surgical Decision Rule
Subglottic Tumor Extension [37]	>15 mm subglottic spread	High likelihood of thyroid gland involvement → consider total laryngectomy and evaluate need for thyroidectomy
Anterior Commissure Thickness [36]	Thickened or asymmetric commissure	Commissural thickening or asymmetry should prompt careful correlation with endoscopic findings, imaging quality, tumor extent, and surgeon experience; it may influence margin planning and treatment selection but should not by itself be considered a contraindication to transoral laser microsurgery.
Conus Elasticus Invasion [35]	Tumor crossing inferior glottic boundary	High probability of cricoid or subglottic invasion → wider margins or total laryngectomy required

**Table 3:** Anatomical Barriers and Their Surgical Implications.

Anatomical Barrier/Structure	Morphometric or Structural Feature	Surgical/Oncologic Implication
Conus Elasticus [35]	Fibroelastic membrane forming the lower boundary of the glottis	Tumor crossing this barrier often invades the cricoid or subglottis → may require wider margins or total laryngectomy
Quadrangular Membrane [1]	Supraglottic fibroelastic sheet	Acts as a temporary barrier; involvement indicates supraglottic spread and affects partial laryngectomy feasibility
Pre-epiglottic Space [2]	Fat-filled deep space anterior to the epiglottis	Conduit for occult tumor extension; involvement often contraindicates transoral laser microsurgery and favors open approaches
Paraglottic Space [1,2,35]	Deep compartment lateral to the glottis	Important contributor to staging and treatment planning, but not an independent mandate for total laryngectomy or chemoradiation. Anterior paraglottic involvement, adjacent to the anterior commissure, thyroid cartilage, and anterior glottic–supraglottic interface, mainly raises concern for anterior transcartilaginous extension, commissural margin planning, and feasibility of transoral or open partial approaches. Posterior paraglottic involvement may be more clinically consequential when it approaches the arytenoid, cricoarytenoid unit, posterior paralaryngeal space, or cricoid framework, because it can affect vocal fold mobility, airway protection, posterior margin control, and the functional safety of organ-preservation surgery. Therefore, anterior and posterior paraglottic spread should be reported separately whenever possible and interpreted together with cartilage integrity, cricoarytenoid fixation, subglottic extension, swallowing/airway function, and multidisciplinary treatment goals.
Anterior Commissure [36]	Thin region where vocal folds meet anteriorly	Thickness predicts thyroid cartilage infiltration; key for planning transoral laser microsurgery margins
Broyles’ Ligament [40]	Fibrous band anchoring the anterior commissure	Recognized weak point → tumor easily penetrates into thyroid cartilage; requires meticulous preoperative imaging
Thyroid Cartilage [30]	Sex-dependent angle and lamina thickness	Invasion changes staging from T2/T3 to T4; determines need for total laryngectomy
Cricoid Cartilage [37]	Only complete ring of airway; prone to chondrolysis	Subglottic extension >15 mm or cricoid erosion predicts thyroid gland involvement and mandates more radical surgery
Posterior Paralaryngeal Space [29]	Deep compartment behind the larynx	MRI essential for detecting posterior spread; involvement reduces feasibility of organ-preservation surgery
Pediatric Subglottis [19]	Narrowest airway point in children	Determines graft size for laryngotracheal reconstruction; critical for stenosis surgery

**Table 4:** Morphometry-Linked Interdisciplinary Decision Points in Laryngeal Surgery.

Team Member/Specialty	Morphometry-Dependent Responsibilities	AI-Enhanced Contributions	Impact on Surgical Decision-Making
Otolaryngologist (ENT Surgeon) [29,49,52,53,54]	Interprets VFL, glottic width, thyroid angle, commissure thickness; correlates morphometry with tumor extent and airway geometry	Uses AI-segmented CT/MRI for precise margin planning; relies on AI lesion detection for early cancer identification	Determines feasibility of TLM, partial laryngectomy, or total laryngectomy; selects reconstructive strategy
Radiologist [24,25,29,30,38,47,54]	Extracts morphometric parameters using CT/MRI, including subglottic AP diameter, cartilage integrity, and deep space dimensions	Applies radiomics and AI subsite mapping to differentiate infiltration from inflammation	Provides staging-critical information and identifies deep-space invasion that may shift treatment toward radical surgery
Anesthesiologist [19,21,33,41,42,53,55]	Evaluates airway morphometry, including subglottic diameter, cricoid size, and pediatric airway position, for intubation strategy	Uses AI-graded stenosis and airway lumen quantification to anticipate difficult airway scenarios	Chooses fiberoptic intubation, high-flow nasal oxygen, or alternative airway strategies for safe shared-airway surgery
Pathologist [18,25,35,36,38,53,54]	Assesses margin morphometry, cartilage invasion, and anterior commissure involvement	Integrates AI-assisted histologic classification and margin quantification	Confirms adequacy of resection and guides the need for adjuvant therapy
Medical Oncologist [2,14,24,25,38,47,54,56]	Uses morphometric staging, including deep-space invasion and cartilage involvement, to determine systemic therapy indications	Relies on radiomics-based prognostic models for recurrence risk stratification	Decides on induction therapy, adjuvant chemotherapy, or organ-preservation protocols
Radiation Oncologist [24,25,38,40,47,53,54]	Considers morphometric tumor volume, cartilage invasion, and airway geometry for dose planning	Uses AI-derived tumor segmentation for precise target delineation	Determines suitability of organ-preservation radiotherapy versus surgical intervention
Speech-Language Pathologist [5,9,23,26,49,51,57]	Evaluates functional morphometry, including vocal fold dynamics, mucosal wave, and airway protection	Uses AI-based stroboscopic analysis to quantify glottic insufficiency	Recommends rehabilitation or secondary interventions such as injection laryngoplasty
Neurologist [26,49,50,51,57,58,59,60]	Assesses neuromuscular contributors to morphometric changes, including atrophy and asymmetry	Applies AI-based voice pattern recognition for early detection of neurologic dysphonia	Guides botulinum toxin therapy, neuromuscular rehabilitation, or surgical adjuncts
Biomedical Engineer [5,7,20,45,48,53]	Designs patient-specific implants using CT-derived morphometry, including thyroid lamina geometry and cartilage defects	Uses AI-enhanced 3D reconstruction and photogrammetry for implant modeling	Produces customized titanium or 4D polymer prostheses and improves surgical precision
Data Scientist/AI Specialist [5,20,49,52,53,54,56,61]	Integrates morphometric datasets with radiomics and videomics	Develops segmentation models, prognostic algorithms, and surgical simulation tools	Enhances multidisciplinary decision accuracy and supports predictive personalized treatment pathways

**Table 5:** Imaging Modalities and Their Morphometric Strengths.

Imaging Modality	Morphometric Strengths	Clinical Utility
Computed Tomography [25,30,31,33,36,37,47]	Parallel-plane alignment with vocal folds eliminates geometric distortion Precise measurement of VFL, glottic width, thyroid angle, cricoid/subglottic diameters Accurate assessment of anterior commissure thickness	Standard for preoperative morphometry in cancer and stenosis Predicts thyroid cartilage invasion and subglottic extension
Magnetic Resonance Imaging (MRI) [14,24,25,29,38,47]	Superior soft-tissue contrast Precise mapping of submucosal tumor spread Differentiates tumor infiltration vs. inflammation High accuracy for subsites: pre-epiglottic, paraglottic, posterior paralaryngeal spaces	Optimizes staging for organ-preservation surgery Critical for evaluating cartilage involvement in T3 tumors Guides decisions between partial vs. total laryngectomy
Ultrasonography [26,49,51]	Real-time measurement of vocal fold length and dynamics Non-invasive, radiation-free	Monitoring progression of voice disorders

**Table 6:** Future Proposal of AI-Driven Decision Rules in Laryngeal Surgery.

AI Capability	Quantitative Performance/Functional Insight	Surgical Decision Rule
CNN-based Endoscopic Lesion Detection [6,17,22,53,62]	Sensitivity 71–98%, specificity 86–98% for early cancer detection	High-confidence identification of suspicious mucosal lesions → prioritize biopsy or early TLM (transoral laser microsurgery)
AI-Assisted Differentiation of Benign vs Malignant Lesions [6,17,22,52,53,54,62]	Videomics + radiomics improve discrimination of early LSCC	When AI flags malignant probability above threshold → avoid conservative excision; plan oncologic resection
Automated Vocal Fold Segmentation (CT/MRI) [4,5,16,30,31,33,49,53]	Objective extraction of VFL, glottic width, thyroid angle	Reduces manual error → use AI-derived morphometry for preoperative planning in partial laryngectomy and thyroplasty
AI-Enhanced Mapping of Subsite Invasion (MRI) [14,24,25,29,38,47,53]	Accurate delineation of pre-epiglottic, paraglottic, posterior paralaryngeal spread	AI-assisted mapping of suspected deep-space invasion may support segmentation, detection, and multidisciplinary review; treatment selection should remain based on integrated radiologic, endoscopic, pathological, functional, and patient-specific assessment rather than AI output alone.
Radiomics-Based Prediction of Recurrence/Survival [25,38,47,52,53,54,56]	Multimodal radiomics + deep learning outperform clinical staging	High-risk radiomic signatures may support prognostic assessment and multidisciplinary discussion, but adjuvant therapy should currently be determined by established clinicopathological risk factors, such as margin status, extracapsular extension, nodal disease, perineural or lymphovascular invasion, and pathological stage, rather than radiomic features alone.
AI-Driven Quantification of Anterior Commissure Involvement [30,31,35,36,40,47,52,53]	Precise measurement of commissure thickness and asymmetry	AI-detected commissure infiltration → avoid TLM; consider open partial laryngectomy
AI-Derived Subglottic Extension Measurement [30,31,33,37,47,52,53,54]	Automated AP diameter and extension quantification	AI-measured extension >15 mm → predict thyroid gland involvement; plan total laryngectomy
Videostroboscopic AI (Mucosal Wave Analysis) [5,23,26,49,51,57]	Objective vibratory metrics: symmetry, periodicity, amplitude	AI-detected glottic insufficiency → consider injection laryngoplasty or medialization thyroplasty
Photogrammetry-Based 3D Reconstruction [5,7,20,45,48,53]	High-fidelity geometry from endoscopic video	AI-generated 3D model → simulate surgical approach and optimize resection margins
AI-Assisted Stenosis Grading [16,19,33,41,42,53,55]	Quantitative airway lumen measurement	Severe stenosis detected → plan cricotracheal resection or laryngotracheal reconstruction

**Table 7:** Proposal of Integrated Morphometry with AI Surgical Workflow.

Workflow Stage	Morphometric Input	AI Contribution	Surgical/Clinical Decision Output
1. Initial Endoscopic Assessment [6,17,22,36,49,53,62]	Visual estimation of lesion size, anterior commissure involvement, glottic width	CNN-based lesion detection (71–98% sensitivity) flags suspicious mucosal areas; videomics quantifies mucosal wave	Early biopsy prioritization; selection of TLM vs. conservative excision
2. Baseline Morphometric Mapping (CT/MRI) [30,31,33,36,37,49,52,53,54]	VFL, thyroid angle, cricoid diameter, subglottic AP diameter, commissure thickness	AI segmentation extracts precise measurements; radiomics identifies high-risk patterns	Personalized surgical planning: partial vs. total laryngectomy; airway management strategy
3. Deep Space Evaluation (MRI) [14,24,25,29,38,47,53]	Pre-epiglottic, paraglottic, posterior paralaryngeal space dimensions	AI subsites mapping differentiates infiltration vs. inflammation	Organ-preservation feasibility assessment; decision for open vs. endoscopic approach
4. Tumor Extension Quantification [25,30,35,37,47,54]	Subglottic extension length; cartilage integrity	AI quantifies extension; flags >15 mm threshold predicting thyroid gland involvement	Decision for thyroidectomy, extended resection, or total laryngectomy
5. Airway Morphometry (Adults & Pediatrics) [16,19,33,41,42,53,55]	Adult subglottic diameter; pediatric narrowest airway point	AI stenosis grading; automated lumen measurement	Selection of graft size, cricotracheal resection planning, endotracheal tube sizing
6. Functional Morphometry (Ultrasound/Stroboscopy) [5,23,26,49,51,57]	Vocal fold dynamics, symmetry, amplitude	AI mucosal wave analysis detects glottic insufficiency	Decision for injection laryngoplasty, medialization thyroplasty, or rehabilitation
7. 3D Surgical Simulation [5,7,20,45,48,53]	CT-derived laryngeal geometry; thyroid lamina dimensions	AI-enhanced photogrammetry reconstructs 3D anatomy; predicts resection margins	Preoperative simulation; design of patient-specific implants
8. Prognostic Modeling [25,30,38,47,52,53,54,56]	Tumor morphotype, cartilage invasion, airway geometry	Radiomics + deep learning predict recurrence and survival	Intensification of adjuvant therapy; MDT risk stratification
9. Postoperative Monitoring [9,23,26,49,51,52,53,54]	Longitudinal morphometric changes; airway remodeling	AI tracking of lesion recurrence and airway narrowing	Early detection of recurrence; personalized rehabilitation trajectory

**Table 8:** Age- and Sex-Specific Morphometric Norms.

Parameter	Sex-Specific Norms	Age-Specific Norms	Clinical Implications
Vocal Fold Length (VFL) [3,4,16,33,39,51]	Longer and wider in males; shorter in females	Decreases subtly with age due to muscle atrophy	Determines pitch, informs phonosurgery and partial laryngectomy planning
Internal Thyroid Cartilage Angle [3,4,16,39,43,46]	Markedly narrower in males; wider in females	Angle may widen slightly with cartilage remodeling in older adults	Critical for thyroplasty, predicting VFL, and shaping laryngeal geometry
Thyroid Lamina Height & Width [3,4,7,16,39,45]	Larger dimensions in males; smaller in females	Age-related ossification alters contour after ~60 years	Predicts VFL; used for surgical planning and 3D implant design
Cricoid Sagittal Diameter [3,4,16,33,41,42,55]	Significantly greater in males	Cartilage calcification increases rigidity with age	Guides endotracheal tube sizing and stenosis surgery
Subglottic Diameter (Adults) [3,4,16,33,42,55]	Larger in males	May narrow with age due to cartilage ossification	Essential for stenosis management and airway reconstruction
Subglottic Diameter (Pediatrics) [19,20,41,42,44]	Minimal sex differences pre-puberty	Narrowest airway point in infants and children; larynx positioned at C3–C4	Determines graft size for pediatric laryngotracheal reconstruction
Laryngeal Position [19,41,42,44,55]	No major sex difference	Pediatric larynx at C3–C4; adult larynx descends to C5–C6	Influences airway management and surgical exposure
Cartilage Ossification [3,16,39,43,46,47]	Similar pattern in both sexes, but earlier/more pronounced in males	Ossification increases after age 60; up to 54% prevalence	Prevents misinterpretation of imaging; affects surgical rigidity
Hyoid-Larynx Complex Fusion [3,39,43,44,46]	No major sex difference	Complete bilateral fusion of hyoid body and greater horns in ~25% of adults	Alters biomechanics of swallowing and phonation
Post-Pubertal Laryngeal Shape [3,4,16,34,39,43]	Male larynx becomes larger and more angular; female larynx remains smaller and rounder	Shape influenced by age, sex, BMI	Affects mechanical behavior during phonation and airway protection

## Data Availability

No new data were created or analyzed in this study. Data sharing is not applicable to this article.

## Abbreviations

The following abbreviations are used in this manuscript:

3D	Three-dimensional
4D	Four-dimensional
AI	Artificial intelligence
AP	Anteroposterior
BMI	Body mass index
CNN	Convolutional neural network
CT	Computed tomography
ENT	Ear, nose, and throat
MDT	Multidisciplinary team
MeSH	Medical Subject Headings
MRI	Magnetic resonance imaging
TLM	Transoral laser microsurgery
VFL	Vocal fold length
VHI	Voice Handicap Index
